# Age Differences in Emotion and Coping During the COVID-19 Pandemic

**DOI:** 10.1093/geroni/igab046.2087

**Published:** 2021-12-17

**Authors:** Joseph Mikels, Nathaniel Young, Alyssa Minton

**Affiliations:** DePaul University, Chicago, Illinois, United States

## Abstract

The COVID-19 pandemic unleashed a relentless stressor on the human species with numerous deadly risks. These risks have been disproportionately threatening to the health and wellbeing of older adults. Since April 2020, we have been studying how the pandemic has affected the emotional experiences of older and younger adults broadly in several studies. For instance, in one study, we found that older adults (N=176) experienced fewer negative emotions and coped with greater levels of agency than younger adults (N=181). In additional work, we have been examining how these age differences differ for older workers versus retirees as well as in minority populations. This work broadly supports and illuminates our recent theoretical framework that focuses on how evaluative appraisal processes underlie and contribute to age differences in emotional experience generally, but especially in the context of the stress experienced during a global pandemic.

